# Laparoscopic Management of Initially Unrecognized Splenic Hydatid Cysts: A Case Report and Review of the Literature

**DOI:** 10.3390/medicina55120771

**Published:** 2019-12-03

**Authors:** Vladimir Milosavljevic, Milan Veselinovic, Boris Tadic, Danijel Galun, Miljan Ceranic, Dragan Eric, Milos Bjelovic

**Affiliations:** 1 General Hospital “Stefan Visoki”, 11420 Smederevska Palanka, Serbia; milosavljevicvladimir10@gmail.com; 2 Clinical Centre of Serbia, Clinic for Digestive Surgery, 11000 Belgrade, Serbia

**Keywords:** hydatid disease, echinococcus granulosus, cyst, spleen, laparoscopy, splenic cyst

## Abstract

We present a case report that demonstrates diagnostic and intraoperative challenges in the laparoscopic management of initially unrecognized splenic hydatid disease. A male patient, aged 44, was admitted to our department with a big unilocular splenic cyst, radiologically (ultrasonography, computed tomography) characterized as a simple cyst. Serological tests for anti-Echonococcus antibody were negative, and chests X-ray findings were unremarkable, so laparoscopic cyst fenestration with omentoplasty was planned. The intraoperative finding did not correspond to a simple splenic cyst. Hydatid daughter cysts were recognized after the careful opening of the cyst wall. The operation was completed without shifting to open procedures. Laparoscopic partial pericystectomy with omentoplasty is a safe and effective surgical procedure for the management of splenic hydatid disease.

## 1. Introduction

Hydatid disease is a systemic zoonotic disease caused by tapeworms of the genus *Echinococcus*. Infection in humans occurs accidentally by the ingestion of parasite eggs excreted in dog feces through hand-to-mouth transfer. Although splenic hydatid disease is a rare clinical entity with a prevalence of 2.5%–5.8%, the spleen is, after the liver and lungs, the third most common organ involved [1]. Depending on the stage of the disease, a hydatid cyst in the spleen can share very similar imaging findings with the splenic cysts of other etiology [2]. Even with the use of modern radiological imaging techniques, this form of hydatid disease is a rare occurrence and may pose a diagnostic challenge. Surgical management employs a wide range of interventions, from splenectomy to organ-sparing surgical procedures [3,4,5].

In this paper, we want to share our experience in the management of an initially unrecognized splenic hydatid cyst, with an idea to represent laparoscopic partial pericystectomy as a safe and effective surgical procedure for the management of splenic hydatid disease. This study was approved by the Ethics Committee of the Clinical Centre of Serbia No. 3098/39 (date of approval 18 January 2019). Written informed consent was obtained from the patient.

## 2. Case Report

A male patient, age 44, was admitted to the Clinic for Digestive Surgery within the Clinical Center of Serbia on 15 December 2015 due to dull abdominal pain and bloating under the left rib cage. His medical history was not remarkable. On physical examination, we found a big painless, palpable mass on the left side of his abdomen. He was afebrile with normal vitals, and no associated nausea, vomiting, or fever was present. Laboratory examinations, including complete blood count, were within normal ranges, except for mild leucocytosis. Tumor markers (CA 19–9, CEA, AFP) were all unremarkable. Biochemistry test results showed some features of the chronic inflammatory response through moderately elevated C-reactive protein and fibrinogen level, 34.6 mg/L and 4.8 g/L, respectively.

**Figure 1:**
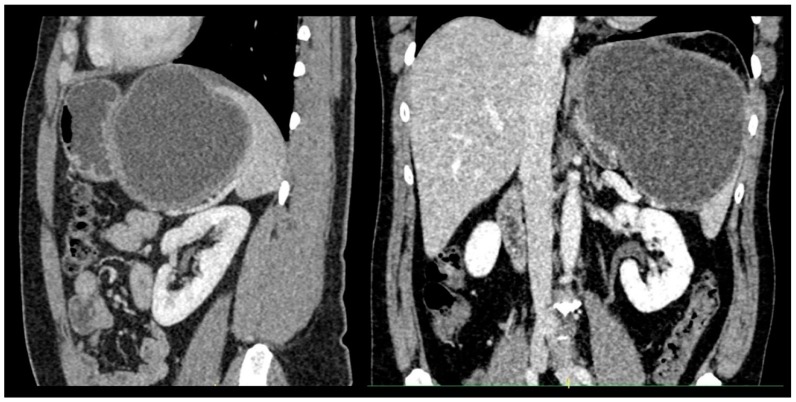
Abdominal computed tomography showing a large splenic cyst.

Abdominal ultrasonography and computed tomography (CT) revealed an unilocular 12 cm splenic cyst (Figure 1). It was characterized as a simple splenic cyst. As our country is an endemic area for echinococcosis, we performed serological tests for anti-Echonococcus antibody, and they were negative. Chests X-ray findings were also unremarkable. 

In accordance with the preoperative imaging findings, we opted for laparoscopic cyst fenestration with omentoplasty. The patient was preoperatively evaluated by a pulmonologist and cardiologist, and adequately prepared for surgical procedure.

**Figure 2:**
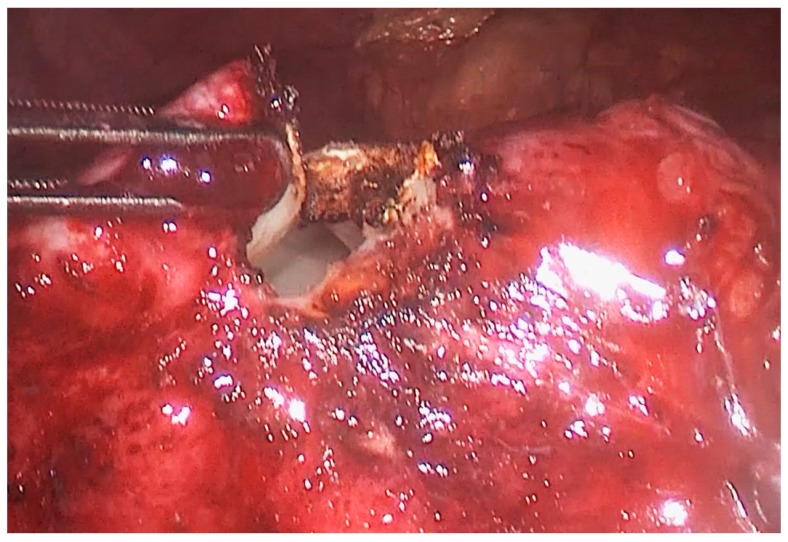
Intraoperative photo: Cystic lesion with a thickened wall.

**Figure 3:**
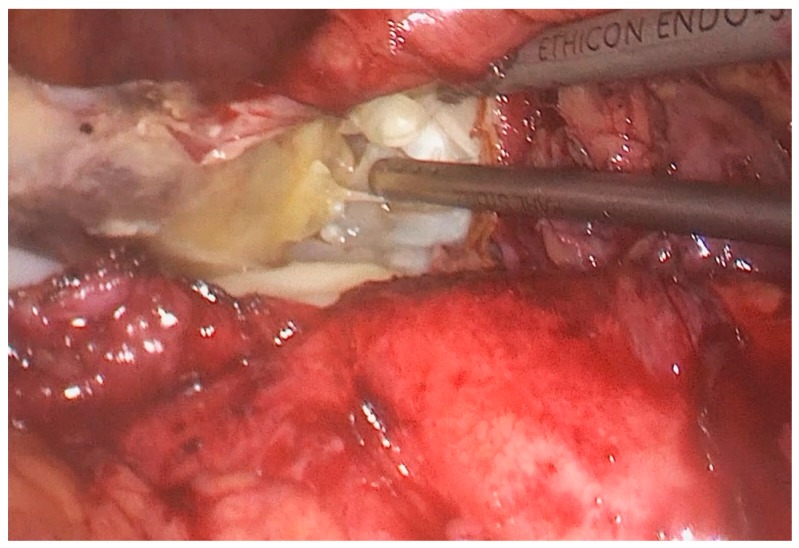
Intraoperative photo: daughter cysts in the hydatid cyst cavity.

**Figure 4:**
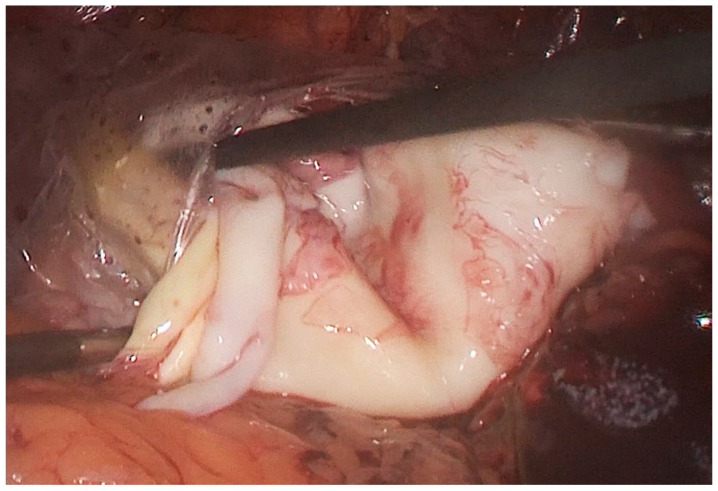
Intraoperative photo: Germinative membrane placed in a polyethylene bag.

**Figure 5:**
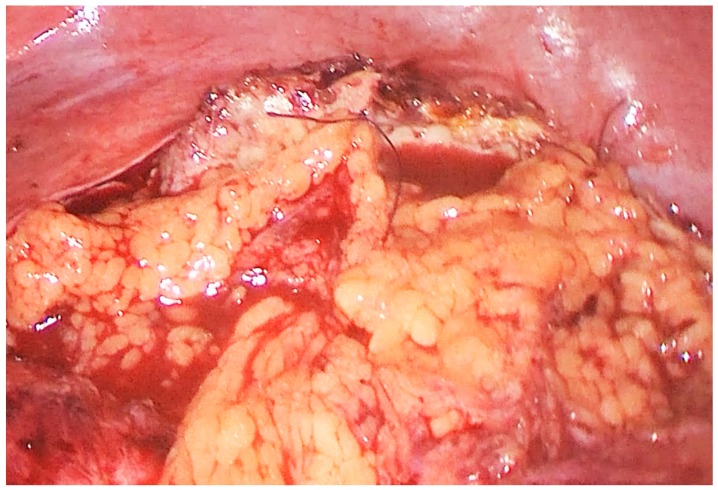
Intraoperative photo: Omental patch placed in the cyst cavity.

The patient was placed in the right lateral position, i.e., the so-called “hanging spleen” technique [6]. Laparoscopic exploration verified the splenic cystic lesion with thickened wall and segment of greater omentum adherent to the cyst, i.e., the intraoperative findings did not correspond to a simple splenic cyst (Figure 2). Omental adhesions were sharply removed from the cyst by a laparoscopic dissector. After the careful opening of the cyst wall using a laparoscopic harmonic scalpel (Ultracision^®^, Ethicon Inc., Somerville, NJ, USA), daughter cysts were found (Figure 3), so we realized that it was, in fact, an unrecognized splenic hydatid cyst. Intraoperative consideration of shifting into an open procedure was compromised by a possible risk of intraperitoneal spilling of cystic content, so we decided to complete surgery laparoscopically. Gauzes soaked with 20% saline were immediately packed in the operative field to avoid the dissemination of the parasite during surgery. Hydatid liquid was aspirated by the laparoscopic suction-irrigation device (Sclartech^®^, Sclar Instruments, West Chester PA, USA) as well as the daughter cysts. The germinative membrane was completely removed by laparoscopic forceps, placed directly in the polyethylene bag (Endopouch retriever^®^, Ethicon Inc., Somerville, NJ, USA) and extracted from the abdomen (Figure 4). The cavity of the cyst was flushed with 20% saline solution. After the scolicidal agent was aspirated, a wide excision of the hydatid cyst wall, a so-called partial pericystectomy, was performed with a laparoscopic harmonic scalpel to prevent and control bleeding from splenic tissue. Finally, we performed omentoplasty. After separation from the transverse colon and division of some gastroepiploic vessels, an omental flap was developed, transposed into the cystic cavity, and fixed with few sutures to the edge of the cyst. (Figure 5).

**Figure 6:**
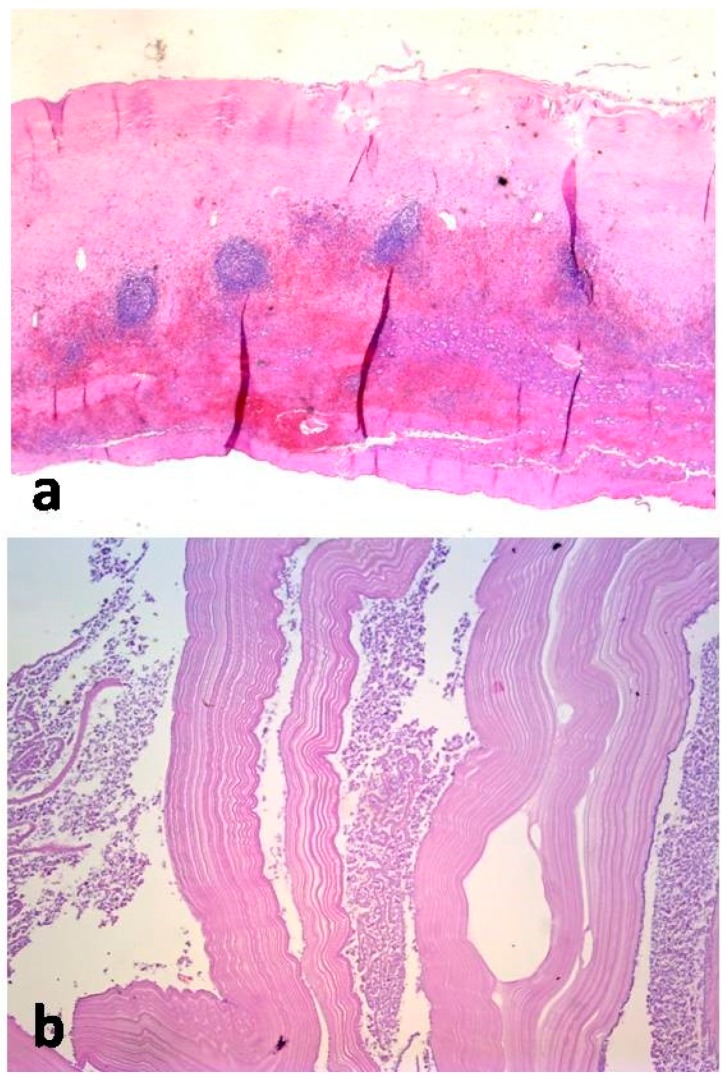
Histological examination: (**a**) Cystic lesion revealed partly hyalinized inner densely fibrous lining and outer layer infiltrated with lymphocytes, eosinophils, and neutrophils associated with some elements of granulation tissue. (**b**) Sample of cystic content, was consistent with the laminated cuticle of the hydatid cyst and some “hydatid sand”, although parasitic scolices were not found.

Histological examination of the cystic membrane revealed partly hyalinized inner densely fibrous lining and outer layer infiltrated with lymphocytes, eosinophils, and neutrophils associated with some elements of granulation tissue. A sample of cystic content, which was separately submitted, was consistent with the laminated cuticle of the hydatid cyst and some “hydatid sand”, although parasitic scolices were not found (Figure 6).

The postoperative recovery went very well and the patient was discharged from hospital on the third postoperative day. To prevent recurrence of the disease, Albendazole therapy was prescribed in two cycles of 4 weeks, each with a break of 2 weeks between each cycle. The patient was followed every six months for four years by abdominal ultrasound and abdominal and pelvic CT. There has been no evidence of recurrence.

## 3. Discussion

The hydatid splenic cyst is a rare clinical entity with a prevalence of 2.5%–5.8% [1]. It was first reported by Berlot in 1790 [7].

There are few mechanisms explaining the parasitic disease in the spleen. The eggs of the parasite can escape the lung and liver barrier before entering the systemic circulation and setting in the spleen [8]. There is also a possibility of retrograde spread via the portal and splenic vein. After leaving the digestive tract and crossing to portal circulation, parasites continue to retrograde through the portal and lienal vein to spleen parenchyma. Also, splenic involvement may result from gastric or colonic trans-parietal passage of eggs or intraperitoneal rupture of a hepatic hydatid cyst [9].

The diagnosis of this disease is based on the combination of clinical presentation, serologic tests, and imaging. The commonly used serologic tests are ELISA with a sensitivity of more than 90%, the test of indirect haemagglutination (sensitivity of 85%), and immunoelectrophoresis (sensitivity of 90%) [1,8]. Although hydatid cysts can be described with precision by means of modern radiological diagnostics, abdominal ultrasound is still the cornerstone of diagnosis, staging, and follow-up (sensitivity of 90%–95%). Ultrasonographic classification of hydatid cysts and the estimation of their viability is based on Garbie’s classification [10]. Despite the high specificity and sensitivity of diagnostic procedures, about 10% of cases remain unrecognized [11]. Splenic lesions such as simple cysts, pseudocysts, solitary abscesses, and hematomas are the most common differentials [12]. Considering radiologically clearly visible calcifications (Gharbi type III and type IV) and detached germinative membrane (Gharbi type II), we assume that most of the unrecognized splenic hydatid cysts are Gharbi type I as they have a quite similar radiological appearance as simple cysts which are the commonest type of splenic cyst [13].

There are several treatment modalities for splenic hydatid disease, such as the use of antihelmintic drugs, percutaneous drainage of the hydatid cyst, and surgery. 

Surgical management is considered to be superior since it provides good long-term results with minimal complications. It employs a wide range of procedures, from splenectomy, either through open or laparoscopic approaches, to organ-sparing surgery including partial splenectomy, cyst enucleation, or partial pericystectomy [4,5].

The minimally invasive approach has become the gold standard for the surgical treatment of most splenic conditions. Some authors advocate complete splenectomy, reducing the possibility of recurrent disease and reducing postoperative bleeding, but most authors recommend a laparoscopic approach, especially for superficial, easily accessible cysts [14,15]. The justification for this attitude stems from the fact that the laparoscopic approach, compared to open surgery, is a minimally invasive procedure providing quick recovery while being safe and effective [16,17,18]. A statistically significant difference, in terms of postoperative complications and recurrence of the disease, has been evidenced by comparing the results of total splenectomy and organ-sparing surgical procedures. Priority is given to an organ-sparing surgery, i.e., spleen preservation, considering its important immunological function, especially in children.

Laparoscopic fenestration with excision of the part of the wall represents an effective and definitive treatment for most of the splenic cysts [13]. However, this modality of treatment requires special caution in endemic areas where a higher prevalence of splenic echinococcus can be expected. Laparoscopic manipulations must be as gentle as possible alongside the careful opening of the cyst. A careless opening of the cyst and uncontrolled spillage of cyst’s content from an unrecognized splenic hydatid cyst can, along with elevated intracystic pressure, lead to life-threatening complications like anaphylaxis or seeding (secondary echinococcosis) into peritoneal cavity. The surgeon should have adequate skills and experience in laparoscopic surgery to manage such an unexpected situation.

Jones et al. in 1992 published the first report about laparoscopic surgery of hydatid disease in 1992 [19]. Searching bibliographic data, we found numerous reports about hydatid disease managed laparoscopically but focusing mainly on hepatic hydatid disease. Studies describing the laparoscopic approach in the management of splenic hydatid disease are limited to case reports, while head-to-head studies comparing laparoscopic and open approaches are not available. However, studies on hepatic hydatid disease indicate that the laparoscopic approach is a safe and efficient treatment modality. Our study indicates that a laparoscopic approach may be employed in the management of splenic hydatid disease, although more studies are needed to confirm its safety and feasibility.

## 4. Conclusions

Despite the fact that isolated splenic hydatid disease is a rare condition, it should be considered in the evaluation of splenic cystic lesions, especially in endemic areas. Although serological testing, abdominal ultrasound, and CT are characterized by high specificity, the diagnosis of a sasplenic hydatid cyst may be a diagnostic challenge. Gauze packing and scolicidal agents should be disposed of and prepared preoperatively. Careful laparoscopic cyst examination, a laparoscopic suction device inserted into the abdomen, and a small cyst opening should be followed by the gauze packing of the operative field to avoid dissemination of the parasite during surgery in case of splenic hydatid disease. The procedure should be performed by an experienced surgeon with advanced laparoscopy skills, collaborating theatre staff, and appropriate equipment.

An interesting question can arise: will the minimally invasive surgical approach overcome conventional surgery and become the standard modality for splenic hydatid disease treatment? This case report indicates that the laparoscopic partial pericystectomy can be a safe and efficient treatment modality.
